# Large Kidney Cysts in *HNF1B* Nephropathy Mimicking Autosomal Dominant Polycystic Kidney Disease

**DOI:** 10.1177/20543581241232470

**Published:** 2024-02-15

**Authors:** Nada Alamri, Matthew B. Lanktree

**Affiliations:** 1 Department of Medicine, McMaster University, Hamilton, ON, Canada; 2 Department of Health Research Methods, Evidence & Impact, McMaster University, Hamilton, ON, Canada; 3 Population Health Research Institute, Hamilton, ON, Canada; 4 Division of Nephrology, St. Joseph’s Healthcare Hamilton, Hamilton, ON, Canada

**Keywords:** HNF1B-related kidney disease, kidney cysts, genetic diagnosis, ADPKD, HNF1Beta nephropathy

## Abstract

**Rationale:**

Hepatocyte nuclear factor 1 beta (*HNF1B*) nephropathy is a rare autosomal dominant monogenic kidney disease. We present a case mimicking autosomal dominant polycystic kidney disease (ADPKD), highlighting the phenotypic heterogeneity of *HNF1B*-related disease.

**Presenting concerns of the patient:**

A 37-year-old man presented with hypertensive urgency, accompanied by flank pain and abdominal distension. Despite the absence of familial kidney disease, imaging revealed large bilateral kidney cysts resembling ADPKD.

**Diagnosis:**

We initially suspected de novo ADPKD. However, negative genetic testing results for *PKD1* and *PKD2* led to a 43-gene cystic kidney sequencing panel which identified a deletion encompassing the entire *HNF1B* gene.

**Intervention:**

To alleviate discomfort caused by the kidney cysts, ultrasound-guided aspiration and foam sclerotherapy were performed. Tolvaptan, used for treating high-risk ADPKD, was not prescribed after confirming the diagnosis was *HNF1B* nephropathy.

**Outcomes:**

A diagnosis of *HNF1B* nephropathy was reached following gene panel testing. Abdominal symptoms improved following cyst aspiration and foam sclerotherapy.

**Novel findings:**

*HNF1B* nephropathy has a variable presentation but can lead to cysts appearing like ADPKD. A 43-gene cystic kidney sequencing panel identified the diagnosis in this uncertain case.

## Abrege

**Justification:**

La néphropathie associée à *HNF1B* est une maladie rénale monogénique autosomique dominante rare. Nous présentons un cas s’étant présenté comme une polykystose rénale autosomique dominante (ADPKD), ce qui met en évidence l’hétérogénéité phénotypique de la néphropathie associée à *HNF1B*.

**Présentation du cas:**

Un homme de 37 ans présentant une crise hypertensive accompagnée de douleurs au flanc et d’une distension abdominale. Malgré l’absence d’antécédents familiaux de néphropathie, l’imagerie a révélé de gros kystes rénaux bilatéraux ressemblant à l’ADPKD.

**Diagnostic:**

Nous avons initialement suspecté une ADPKD *de novo*. Cependant, les résultats négatifs aux tests génétiques pour *PKD1* et *PKD2* ont conduit à un panel de séquençage de 43 gènes de rein kystique qui a permis d’identifier une délétion englobant l’ensemble du *gène HNF1B*.

**Intervention:**

Une aspiration et une sclérothérapie à la mousse guidées par échographie ont été effectuées pour soulager l’inconfort causé par les kystes rénaux. Le tolvaptan, qui est utilisé pour traiter le risque élevé de progression de l’ADPKD, n’a pas été prescrit après la confirmation du diagnostic de néphropathie associée à *HNF1B*.

**Résultats:**

Un diagnostic de néphropathie à *HNF1B* a été posé à la suite du test de panel de gènes. Les symptômes abdominaux se sont améliorés après l’aspiration des kystes et l’échosclérothérapie à la mousse.

**Nouveaux résultats:**

La néphropathie associée à *HNF1B* a une présentation variable, mais peut conduire à l’apparition de kystes comme l’ADPKD. Un panel de séquençage de 43 gènes de rein kystique a confirmé le diagnostic dans ce cas incertain.

## Introduction

Hepatocyte nuclear factor 1 beta (*HNF1B*) nephropathy is a rare autosomal dominant monogenic disease caused by pathogenic variants in the *HNF1B* gene located on chromosome 17q12. The prevalence of *HNF1B* nephropathy remains poorly quantified and underrecognized, but it appears to represent less than 1% of adult patients reaching kidney failure.^1^ *HNF1B* codes for a transcription factor that influences the expression of numerous genes in many tissue types, including uromodulin (*UMOD*), fibrocystin (*PKHD1*), and polycystin-2 (*PKD2*) in the kidney.^2^ Both single nucleotide variants and deletions in *HNF1B*, including whole gene deletions, have been reported as causal of *HNF1B* nephropathy.^3,4^

*HNF1B* nephropathy leads to diverse clinical manifestations including renal cysts and diabetes (RCAD), autosomal dominant tubulointerstitial disease (ADTKD), congenital anomalies of the kidneys and urinary tract (CAKUT), hypomagnesemia, hyperuricemia, and hypocalciuria.^5-7^ The kidney morphology in *HNF1B* nephropathy is usually reported to overlap with medullary sponge kidney or ADTKD (caused by *UMOD, REN, MUC1* variants) with multiple small cortical cysts without kidney enlargement that do not progressively increase in number or size over time (Table 1).^3,8^

**Table 1.** Cystic Kidney Phenotypes Reported in Surveys of HNF1B Nephropathy.

Study	Population	Kidney cyst findings	Kidney enlargement
Heidet et al^4^	377 patients with *HNF1B* variants, including 271 children, 57 adults, and 49 fetuses	Isolated bilateral hyperechogenic kidneys, prenatal onset, cystic hypoplasia, hyperechogenic kidneys with microcysts, unclassified renal cystic dysplasia	No cases of large cystic kidneys were reported. Patients with moderately enlarged hyperechogenic kidneys before birth showed a reduction in kidney growth after birth
Faguer et al^9^	27 adults from 20 families with *HNF1B* variants or deletions	Cystic phenotype observed in 62% of the adult HNF1B population. Most cystic patients had less than 5 cortical cysts in each kidney	Cysts visible on ultrasound, mostly in the cortex; 2 patients with kidney enlargement
Dubois-Laforgue et al^3^	201 patients with *HNF1B* variants	Renal cysts were observed in 81% of cases. Kidney morphological abnormalities in 151 out of 166 patients	Large cystic kidneys not described
Okorn et al^10^	62 children with HNF1B nephropathy	Bilateral dysplasia with cysts in 74% of cases. Unilateral dysplasia with cysts and contralateral kidney agenesis was found in 3%. Unilateral cystic dysplasia with a normal contralateral kidney in 13%. 8% showed renal dysplasia without cysts	No reports of enlarged cystic kidneys

**Figure 1.**
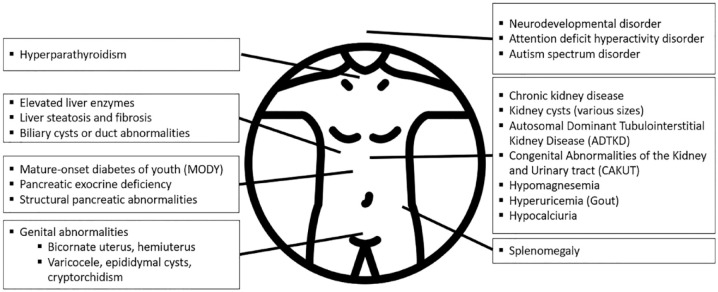
Manifestations of *HNF1B*-related disease.

Additional extra renal manifestations of *HNF1B*-related disease include maturity-onset diabetes of the young (MODY), pancreatic exocrine dysfunction and structural abnormalities, elevated liver enzymes, steatosis and fibrosis, as well as neurodevelopmental abnormalities including attention-deficit hyperactivity disorder and autism spectrum disorder (Figure 1).^11-13^ *HNF1B* only accounts for about 1% of MODY cases, but MODY is present in the majority of *HNF1B* nephropathy cases in adulthood. Chronic kidney disease usually precedes the development of diabetes in *HNF1B*-related disease.^13^ New-onset diabetes after transplantation (NODAT) has also been reported in *HNF1B*-related disease.^14^ Hyperparathyroidism has also been reported but may be secondary to chronic kidney disease.^13^ The presence of neurodevelopmental manifestations including attention-deficit hyperactivity disorder and autism spectrum disorder are present in fewer than 10% of *HNF1B*-related disease cases and are more frequently associated with gene deletions (Table 2).^11^

**Table 2.** Neurodevelopmental Disorders Associated With *HNF1B*.

Study	Population	Neurodevelopmental disorders
Nittel et al^11^	695 patients with *HNF1B* variants from 31 studies	Neurodevelopmental disorders present in 25% of those with deletions vs 6.8% of those with single nucleotide pathogenic variants
Loirat et al^12^	86 children with *HNF1B* variants	3 unrelated cases whole gene *HNF1B* deletions and autism spectrum disorder

The current report presents a case of *HNF1B* nephropathy identified with innumerable large kidney cysts, a presentation that initially appeared to mimic autosomal dominant polycystic kidney disease (ADPKD).

## Case Report

A 37-year-old man presented to the emergency room with headaches and was found to be in a hypertensive urgency. He reported experiencing occasional flank pain and abdominal distension. There were no genital abnormalities. He was previously diagnosed with attention-deficit hyperactivity disorder, anxiety, and autism spectrum disorder from a young age. The patient had been estranged from his father, but his mother had no known kidney disease. There was no known history of kidney failure, aneurysms, or sudden death in his extended family.

On physical examination, the patient was 180 cm tall and weighed 70 kg. His blood pressure was 180/75 mm Hg, and his heart rate was 70 bpm. He had a visibly distended abdomen with palpable organomegaly, but additional cardiovascular, respiratory, and neurological evaluations were normal.

Laboratory results showed elevated serum creatinine (213 µmol/L) and reduced estimated glomerular filtration rate (eGFR; 33 mL/min/1.73 m^2^). Liver enzymes, fasting blood glucose, uric acid, magnesium, potassium, calcium, phosphorus, and parathyroid levels were within their normal ranges. Urinalysis was bland without hematuria or proteinuria.

**Figure 2.**
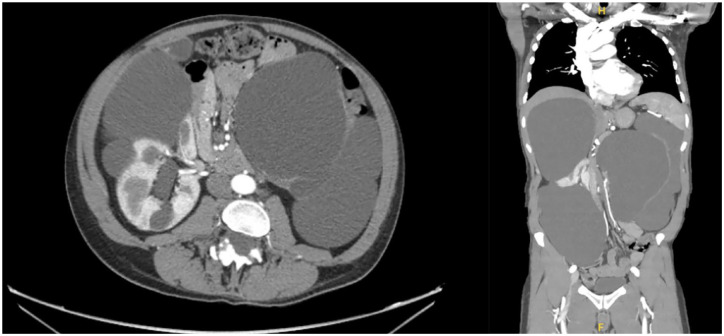
Computed tomography imaging reveals enormous bilateral kidney cysts. There are multiple bilateral extremely large renal cysts, some of which demonstrate thin internal septations without calcifications or nodularity. The cysts are predominantly homogeneous with predominantly fluid attenuation. The largest on the right measures 19 cm in craniocaudal extent. The largest peripheral cyst on the left measures 12 cm. There is resultant mass effect and displacement of the adjacent spleen and liver parenchyma as well as the small bowel mesentery. The spleen is enlarged at 15 cm. The central fluid-filled structure in the left kidney may be a markedly distended collecting system. The left renal parenchyma is markedly thinned and barely perceivable. The right renal pelvis is mildly dilated as well as a moderately distended pole calyx. Both ureters and urinary bladder are within normal limits. The partially visualized aspect of the pancreatic parenchyma is within normal limits.

Computed tomography imaging revealed markedly enlarged kidneys with multiple large bilateral kidney cysts, with the largest cysts measuring 19 cm on the right and 12 cm on the left (Figure 2). The total kidney volume was not reported and was difficult to determine given the abnormal kidney appearance and presence of large exophytic cysts. Some cysts showed thin internal septations, without calcification or nodularity. The spleen was enlarged and measured 15 cm. The left kidney had a markedly distended collecting system, whereas the right kidney pelvis was mildly dilated, with a moderately distended pole calyx. Both ureters were of a normal caliber. The urinary bladder, liver, and the partially visualized aspect of the pancreatic parenchyma appeared normal.

Treatment with amlodipine was initiated and the patient was referred to the McMaster Kidney Genetics Clinic. An initial diagnosis of de novo ADPKD was suspected given typical bilateral morphology and the increase in total kidney volume. Given the negative family history, genetic testing was initiated with sequencing of *PKD1* and *PKD2* which identified no responsible pathogenic variant. A 43-gene cystic kidney sequencing panel (Blueprint Genetics Cystic Kidney Disease Panel, https://blueprintgenetics.com/tests/panels/nephrology/cystic-kidney-disease-panel/) was ordered which identified a heterozygous 1.26 Mb deletion (chr17: g.34842466_36104935del), encompassing the entire *HNF1B* gene. Cascade screening showed his mother did not carry the variant.

Due to the abdominal distention and discomfort caused by the large kidney cysts, the patient was referred to interventional radiology for an ultrasound-guided aspiration and foam sclerotherapy. Two large cysts from each of the right and left kidneys were aspirated removing 2 liters of fluid and 10 milliliters of 2% sodium tetradecyl sulfate was instilled without post-procedural complications. There was an obvious reduction in abdominal distension post-cyst aspiration and sclerotherapy. Repeat imaging is pending to determine whether the fluid in the cysts will recur.

There was a notable shift in the patient’s affect during the diagnostic odyssey. Previous encounters with doctor’s had been centered around his autism spectrum disorder. He was clearly happy that we had been able to obtain a concrete diagnosis that explained multiple manifestations and were able to obtain a treatment that improved his symptoms. The patient’s eGFR has been stable at 30 mL/min/1.73 m^2^ for the last 2 years and blood pressure control is greatly improved. The patient consented to us sharing his story in educational lectures and the current manuscript, and our therapeutic relationship continues.

## Discussion

We describe a patient exhibiting extraordinarily large kidney cysts and kidney enlargement, a clinical picture commonly associated with ADPKD with a high risk for rapid progression to kidney failure. In hindsight, the autism spectrum disorder and dilated collecting system were clues to *HNF1B* nephropathy. However, the markedly large cysts and appearance of kidney enlargement, with the absence of MODY, hyperuricemia, hypomagnesemia, hypocalciuria, pancreatic exocrine dysfunction, liver abnormalities, or ADTKD appearance that are more typical of *HNF1B* led us to believe the diagnosis was ADPKD with autism, rather than *HNF1B*-related disease. The presence of splenomegaly is not typically reported as manifestation of *HNF1B*-related disease, but was present in our patient and has been reported before.^15^

After targeted testing of *PKD1* and *PKD2* were negative, government funding for a cystic kidney gene sequencing panel was approved that revealed a complete heterozygous deletion of *HNF1B*. While massively enlarged polycystic kidneys were reported in monozygotic twins with heterozygous whole gene deletion of *HNF1B*,^16^ *HNF1B* is not typically listed as a cause of ADPKD.^17^ Genetic sequencing panels are becoming more frequently used and can provide unexpected molecular diagnoses, increasing the number of patients with confirmed genetic diagnoses. Significant phenotypic heterogeneity including variable expressivity and incomplete penetrance is now expected as the rule, not the exception, for monogenic diseases and unusual presentations should no longer be surprising.

Given autosomal dominant inheritance, an affected parent is expected, but similar to other autosomal dominant genetic disorders, as many as 50% of new cases can occur without an identified family history, either the result of unclear family history as in this case, or as a result of de novo variants.^18^ Case identification is challenging as no sign or symptom is pathognomonic of *HNF1B-*related disease. A HNF1β score was published as a rationale to guide genetic screening of suspected cases.^18^ The presence of multiple kidney cysts bilaterally in the absence of an ADPKD diagnosis, as was found in the current case, yields a score of 10 which the authors indicate should trigger a consideration of *HNF1B* screening. The rise of gene panel testing means most patients with kidney cysts will likely undergo *HNF1B* screening going forward.

Large deletions may be missed in whole-exome sequencing, as the break points may not be captured in the exon sequence and the read depth may not be sufficient to identify significantly fewer reads of the deleted sequence.^19^ Gene panels typically have greater read depth and ability to identify deletions than exome sequencing.^20^ Targeted techniques such as multiplex ligation-dependent probe amplification (MLPA) may also successfully identify *HNF1B* deletions.^21^

Foam sclerotherapy was performed to provide symptomatic relief of the mass effect from massive cysts. The procedure was well tolerated with beneficial impact on his abdominal distention and discomfort. Tolvaptan was initially considered based on his clinical presentation of large kidneys with innumerable bilateral cysts, reduced eGFR, and presumptive ADPKD diagnosis, but was not prescribed after the diagnosis of HNF1β nephropathy was obtained. In this case, genetic testing made significant differences to diagnosis, management, and patient satisfaction.

## Summary

Typical HNF1β nephropathy manifestations of small kidneys and interstitial fibrosis, hypomagnesemia, and hyperuricemia were missing in this case, and instead, the large kidney cysts and total kidney size appeared more in keeping with a classical presentation of ADPKD. Features of *HNF1B-*related disease including MODY, pancreatic, genital, and liver abnormalities were also missing, but the presence of autism spectrum disorder provided a clue that may have helped the astute clinician. Gene panel sequencing was required to arrive at the correct diagnosis in this case. *HNF1B* sequencing via a gene panel should be considered in patients with suspected ADPKD but with no responsible pathogenic variant identified.
